# Emergent Change in a Mexican Midwifery Center Organization Amidst the COVID-19 Crisis

**DOI:** 10.3389/fsoc.2021.611321

**Published:** 2021-02-09

**Authors:** Cristina Alonso, Akane Sugimoto Storey, Ilse Fajardo, Hannah S. Borboleta

**Affiliations:** Luna Maya, San Cristobal de las Casas, Mexico

**Keywords:** COVID-19, out-of-hospital birth, midwifery model of care, pregnancy, birth, Mexico, Chiapas, Mexico DF

## Abstract

Luna Maya is a Mexican NGO that operates two full-scope midwifery centers in Mexico City and Chiapas, Mexico, providing woman-centered, culturally appropriate midwifery model maternity care on a sliding cost scale. The COVID-19 health crisis has made it necessary for Luna Maya to quickly incorporate safety protocols for out-of-hospital maternity care. Yet many of the emerging guidelines on maternity care have focused on high-income and hospital settings; there are no specific guidelines for such care in out-of-hospital settings in low- and middle-income countries. Thus we have had to create our own, based on best available and emerging evidence. In this article, we describe the guidelines and protocols we have created in response to COVID-19, the international evidence and recommendations on which we base them, and precisely how we carry them out in practice. We also present and analyze the results of qualitative interviews we conducted for this article with eight of our midwives and eight of our midwifery clients. These interviews reveal the tremendous stresses both midwives and pregnant and birthing women are experiencing as a result of the pandemic, their creative adaptations, and the structural flaws, deficiencies, and inequities of the Mexican healthcare system. The article also addresses Luna Maya’s ongoing challenges in continuing to provide care completely outside of governmental support and in difficult economic times, and demonstrates the extreme need for improvements in the Mexican system of maternity care and for full integration of community-based midwives and out-of-hospital birth.

## Introduction: Placing Catastrophe on Top of a Broken Maternal Healthcare System

The COVID-19 pandemic has highlighted health inequities and systemic gaps and failures (Bambra et al., 2020). Latin America, the most inequitable region in the world (Silva-Peñaherrera et al., 2020), is at the time of this writing (September 2020) home to some of the highest rates of cases (The Guardian, 2020). As a non-governmental and midwife-led organization, Luna Maya operates two midwifery centers (Stevens and Alonso, 2020) in San Cristobal, Chiapas and Mexico City since 2003 to bridge harmful gaps in quality of care and access to midwifery services in Mexico (Alonso et al., 2018). The Luna Maya centers provide full scope midwifery services, both gynecological and obstetric, as well as complementary medicine such as acupuncture, massage, and psychological therapy services. Luna Maya midwives are on staff and provide midwifery care assisted by midwifery students who are learning the Midwifery Model of Care through clinical apprenticeship.

Women who access Luna Maya services are Indigenous, Mestiza (mixed) and foreigners. Services are provided on a sliding scale that enables women of all socio-economic levels to access our services. Payment rates are adjusted by income and subsidized through internal and external funding sources. Women at Luna Maya can choose to birth either at home or at the midwifery center; all other care is provided in the two centers. These two Luna Maya midwifery centers are not integrated into the Mexican healthcare system and therefore rely on private purchasing of equipment and supplies. Similarly, lack of a regulatory body and standards for the practice of midwifery and midwifery centers in Mexico led Luna Maya’s midwifery staff to research, apply, test and refine international protocols to protect themselves and those they serve. Midwives hired by Luna Maya have completed a course of study to follow ICM (International Confederation of Midwives) requirements. Therefore, Luna Maya midwives may also be obstetric nurses, general physicians, professional midwives, or midwives licensed in another country.

Midwives have been recognized as specialists in women's sexual and reproductive health (Sakala and Newburn, 2014) and continue to be on the frontlines of providing care during the COVID-19 pandemic, which has exacerbated existing tensions and failures in full access to safe motherhood as seen through a rights-based perspective (Jolivet et al., 2020). In addition, evidence is mounting that pregnant women and other pregnant people are increasingly seeking out-of-hospital (OOH) care out of fear of C-19 infection in hospitals and fear of violations of birthing rights (Davis-Floyd et al., 2020). Nevertheless, in many settings choices are limited and fear of home birth and lack of possibilities to pay out-of-pocket for midwifery services make this option a very complex one for most women and their families. For over 15 years, Luna Maya has provided a safe ground for women to choose safe and respectful birthing options in a country where cesarean rates exceed 50%, and over 30% of women state that they have experienced obstetric violence (Castro and Frías, 2019). Luna Maya has sustained a cesarean section rate of 15% since its inception in 2004 and has been recognized nationally and regionally as providing a safe space for women to recover their strength and power in birth and health care (Alonso et al., 2018).

As a society and as clinicians, we still lack a clear understanding of the spread and impact of the virus, and healthcare providers have been continuously adapting to ever-changing evidence and recommendations. Initial obstetric recommendations focused on in-hospital settings and high-income countries. Slowly, midwifery associations and OOH specialists have developed more relevant protocols. Luna Maya has been continuously incorporating new ways of working within the pandemic, while still providing women-centered and holistic care. With no official guidelines for OOH/community midwives, many international recommendations have been hard or impossible for them to adopt in low- and middle-income countries such as Mexico.

In this article, we explore what Luna Maya’s two Mexican midwifery centers have learned, and continue to learn, about how COVID-19 affects women's decisions and wellbeing and how these impact their physiologic and psychological processes (ACNM and NACPM, 2012). We describe how the Luna Maya midwives have engaged in a continuous process of protocol adoption, testing, and revision and explore how relationships between women, their families and their midwives are being modified amidst the pandemic through the analysis of a series of qualitative interviews.

Some protocols are here to stay, at least for a while; others might change in the next few weeks, months and certainly years. Certain protocols and recommendations have been impossible or unrealistic to implement; we will describe why. This article includes reflections on changes that midwives have observed in women’s birth experiences, due to the increased stress and hardship brought on by the pandemic and their impacts on women’s psychological and physiologic responses. We also analyze the qualitative interviews we carried out with Luna Maya midwives and clients describing the impact of COVID-19 on their perceptions of choices and options for safe and respectful birth.

## Methods

This article is presented in two discrete sections. The first section, based on our practice experiences, describes how the Luna Maya midwives have adapted existing protocols into clinical practice. We harvested clinical guidelines and protocols from online sources, Facebook groups for midwives and email listservs where maternity care providers were discussing and sharing resources on clinical safety and adaptation during COVID. This section describes how our midwives adapted protocols emerging from high-income settings to Mexico City and Chiapas.

The second section provides insight into the experience of adapting to COVID-19 from the perspective of the midwives who work in Luna Maya and consumers of Luna Maya maternal healthcare services. Using a narrative approach, we conducted qualitative interviews to better understand the complexity of the impacts of COVID and the multi-faceted, non-linear responses to the pandemic during pregnancy and childbirth. Although protocols and clinical guidelines intend to provide frameworks for safe care, the lived experience of midwives adapting to providing care are complex and multi-layered. Similarly, women have had to make decisions based on their perceived risk of COVID-19, as well as the implications of social isolation, perception of risk of public spaces and perceptions of autonomy and control within a global pandemic. Therefore, in order to situate how these protocols and guidelines impacted the experience of birth, it was important to carry out qualitative interviews.

In May 2020, we developed two interview guides stemming from a brainstorming session carried out among the co-authors. The interview guides were revised to ensure a comparative approach between the experience of being a midwife and accessing services before and after COVID-19. Due to the semi-structured nature of the interview guides, interviews were adapted according to participant responses.

Midwives and students within Luna Maya were invited to participate through internal communication mechanisms. Participation was voluntary and one student opted out of participating. We interviewed three midwives and five midwifery students during June 2020. All interviews were carried out using Zoom and recorded.

We also carried out semi-structured qualitative interviews using Zoom with eight women who had accessed services at Luna Maya Chiapas and Mexico City during July 2020. Four women were selected because they had chosen to give birth with Luna Maya *regardless* of the COVID-19 pandemic; two had given birth and two were still pregnant. Four other women were selected because they had chosen to give birth with Luna Maya *because* of the pandemic, of whom two had already given birth and two were still pregnant. Two women are low-income, one from Chiapas and one from Mexico City. Five are middle-income women from Mexico City whose income was severely impacted by the COVID-19 pandemic, causing them to make financial adjustments to their birth plans. One interlocutor is a high-income foreigner living in Mexico City, who was already working from home prior to the pandemic and whose income was not affected by the pandemic.

These eight interlocutors were pregnant or had given birth between April and July of 2020; again, participation was voluntary. These women were selected based upon the criteria described above, including access to a telephone or the internet for a Zoom interview and availability during the interview dates. Three interviews were carried out on the phone and five via Zoom. All interviews were recorded and analyzed using an inductive approach to identify themes and conduct discourse analysis.

## Adapting Global Guidelines to Community Midwifery

COVID-19 was declared a pandemic by the World Health Organization (WHO) on March 11, 2020 (Cucinotta and Vanelli, 2020), yet in Mexico cases remained low and the severity of the outbreak was downplayed. In alignment with other Western countries (Scally et al., 2020), Mexico’s strategy was slow to start and marked with inadequacies that were generated in hopes of mitigating economic impact. An international change of tone prompted Luna Maya to begin a strategy as of March 13, two days after the pandemic was declared and lockdown began in Italy, and as global concern was on the rise. Our initial strategy included measures that were concise, holistic and non-alarmist: signs to advise Luna Maya’s public about COVID-19 symptoms and preventative measures, increased sanitation, a switch to paper hand towels from cloth towels, and an immune support tonic recommended to clients. Yet it quickly became clear that these measures would need upscaling. Families' economies and local supplies were affected early on. As of March 14th, many informally employed individuals were out of work and panic purchasing made it close to impossible to acquire goods such as cleaning products, sanitizers, soaps, medical supplies, protective gear and paper products; food prices were also increasing. These tendencies were exacerbated outside of Mexico City, the nation’s economic and political hub where Mexico’s wealth and infrastructure are concentrated. A semi-organized national strategy with stay-at-home recommendations was implemented by the end of March, with large segments of the population already struggling to make ends meet.

As members of clinical staff of Luna Maya, we co-authors have tracked and evaluated evidence-based recommendations and epidemiological trends as of mid-March to create internal guidelines. Immediate sources reviewed included a National Association of Certified Professional Midwives (NACPM) webinar (NACPM, 2020), the USA’s Centers for Disease Control and Prevention (CDC) (CDC, 2020c), and scientific and news articles. Additional sources examined over the next weeks and months included items from the Royal College of Midwives (RCM, 2020), the World Health Organization (WHO, 2020a; WHO, 2020c), the International Confederation of Midwives (ICM, 2020), Open Access resources, and supplementary OOH midwifery guidelines and resources (CABC, 2020; Dar a Luz Birth and Health Center, 2020; Harper, 2020). Aside from enhanced screening and distancing, early guidance (CDC, 2020b; WHO, 2020c) did not contain precautionary measures essential for midwives, who serve as the guardians of physiologic care for healthy women and newborns (Renfrew et al., 2014), yet highlighted the importance of continued face-to face care for non-COVID-19 conditions, especially for populations at higher risk of delayed care (CDC, 2020a)—hence reinforcing the urgency of a midwifery scope of practice to keep the childbearing continuum as a normal, low risk health experience.

On March 27, the ICM, (2020) urgently called upon governments to consider midwives’ needs, as lack of official guidance translated to lack of PPE allocation for midwives, resulting in otherwise healthy midwives—often with small children and families at home—becoming infected through contact at work and falling ill or dying from COVID-19. The ICM (2020) forewarned the detrimental effects the continued lack of PPE for midwives would have upon the health and wellbeing of women and newborns globally. Midwives have responded by creating their own COVID-19 protocols during the pandemic and have been on the forefront of advocating for superior guidelines that reduce community spread (Tugores and Wiseman, 2020) and include midwives. Now shifting to include midwives’ safety, official guidelines (RCM, 2020; WHO, 2020a) are still oriented towards care that is hospital-based and effectively incorporated into healthcare systems, in high-income contexts. As midwifery centers are not integrated into the Mexican healthcare system (Lopez Arellano et al., 2020; Stevens and Alonso, 2020), each center has modified measures for its unique conditions. Unofficial guidelines shared by midwives and midwifery centers in higher income settings have been key in developing our strategy for Luna Maya. What measures Luna Maya has adopted and how we have implemented these measures will be examined in detail below.

### Masking

Aside from additional cleaning supplies, face masks were among the first items contemplated for Luna Maya’s strategy. For the Chiapas center, many items required shipment, yet it was possible to find local producers of cloth masks, ready for pickup on March 16. Still excluded from official guidance (CDC, 2020b; NACPM, 2020; WHO, 2020a), researchers, midwives, community health workers and the public were already questioning the validity of mask recommendations. Accentuating scarcities, the CDC (2020b) was recommending secured, monitored storage and restricted use of all medical masks, with N95s (WHO, 2020c) for patients with or suspected of having COVID during procedures that might put providers at risk of contact with infectious droplets—uses outside a midwifery scope of practice. Early in the pandemic in Mexico, widespread use of facemasks was undermined by a cultural preference for *not* using them—a preference that carried critical risks (WHO, 2020b).

A few of our midwives expressed unease about spending prolonged hours in close contact with laboring women and family members, prompting Luna Maya to acquire a small supply of disposable medical masks in mid-March for use at births. Even though official recommendations at the time ignored mask wearing, despite evidence gaps, the midwives were clear about the likelihood of respiratory droplet and aerosol dissemination during births and that this could be problematic for them when caring for asymptomatic carriers. Those familiar with birth can easily appreciate the degree of exertion it takes mothers to push babies out and how abundantly splashes and sprays occur with multiple kinds of fluids. In the following weeks, scientific articles began appearing advocating for wide use of non-medical cloth masks and disposable medical masks for all health workers (Abaluck et al., 2020; Greenhalgh et al., 2020; Howard et al., 2020; Tugores and Wiseman, 2020), independent of scopes of practice. By the end of March, Luna Maya was lending cloth masks acquired for midwives to clients during care, and in May began a universal policy requiring mask wearing in both centers for all who enter—despite the fact that Mexico’s leading epidemiologist reiterated that this type of measure was inappropriate. Such inadequate recommendations and communications about C-19, especially for vulnerable and historically excluded sectors of the population, have added fuel to the fire of resistance and backlash in Mexico. Corruption-infused entities like national and local governments have historically obstructed participative engagement and community determination at all levels (Mills, 2017). These tendencies are accentuated in Southern areas like Chiapas, where mistrust of the government runs high due to the social and health inequities suffered by Mexico’s Indigenous populations. During this crisis, multiple communities in Chiapas underserved by governments have ransacked and set fire to their local clinics, hospitals and ambulances out of fear, desperation and anger towards the incapacity of the government to respond to the pandemic and save lives (Mariscal, 2020; Redacción Animal Politico, 2020). In addition, around the country healthcare personnel have been attacked by citizens fearful of providers being contagious or enraged about loved ones who died from COVID-19 (Garcia Bermejo, 2020; Rivers and Gallon, 2020).

A systematic review and meta-analysis specific to SARS-CoV-2 and COVID-19 published in the *Lancet* (Chu et al., 2020), and a WHO change in guidelines in June, prompted a Luna Maya switch in the type of masks used at births. As community transmission escalated in Mexico, low community alignment with national recommendations, evidence on virus aerosolization through vocalization (Asadi et al., 2020; WHO, 2020d), and possibly elevated prevalence of asymptomatic carrier status during pregnancy (Bianco et al., 2020; Sutton et al., 2020) prompted us to acquire higher-filtration, N95-type masks for Luna Maya’s clinical staff to wear during births.

### Eye Protection

Another early measure we considered at Luna Maya was eye protection. Though early guidance was non-specific to midwifery (CDC, 2020a), homebirth COVID-19 guidelines shared among community-based midwives in Mexico and the US made casual mention of its use. Purchasing supplies for upcoming months with emergency funds acquired from Global Force for Healing, Luna Maya included protective glasses. Speculative hypotheses about fecal transmission (WHO, 2020d; Zhang et al., 2020), alongside the abundance of heavy breathing and sprays during labor and birth, prompted us at Luna Maya to recommend routine use of eye protection for our midwives during the second stage of labor, when it is challenging to avoid prolonged close contact, fluids and stool. But the initial protective glasses we obtained clouded easily in combination with facemasks, and were deemed impractical in the kind of low lighting that facilitates spontaneous birth. Midwives tested swimming goggles brought from home, also resulting in poor visibility; thus many births were attended without eye protection.

An RCM (2020) PPE infographic seen on social media by our midwives coincided with intensifying community transmission and fatalities affecting families we serve in Chiapas and Mexico City, and motivated our clinical teams to more closely examine their own PPE implementation and make improvements. Face shields were promptly acquired, resulting in better visibility and a valuable barrier from droplets and splashes. WHO research and guidelines (Chu et al., 2020; WHO, 2020a) published shortly thereafter solidified the appropriateness of this measure. As of early June, we have been recommending regular use of face shields in addition to masks while clinical teams or support staff provide care or do cleaning, administrative work, or use non-individual transportation, especially when spacing and air-exchange ventilation are poor. With local supplies now more readily available, some midwives have also acquired safety goggles.

### Clothing Changes as PPE: Washing in a Middle-Income Setting

Gowns, scrubs or aprons, akin to those normally used in hospital-based care, are commonly substituted in OOH midwifery practice by clean changes of clothing and attentive personal hygiene. It is known that secretions or droplets carrying SARS-CoV-2 can be expelled from infected individuals and contaminate surfaces and objects (WHO, 2020d), creating fomites (inanimate objects or substances capable of transmitting infectious organisms from one individual to another) with viable particles present for 72 h or more (Chin et al., 2020). How long the virus lives on clothing is unknown; nonetheless our clinical teams agreed that a commonsense measure to reduce fomite transmission would be multiple changes of clothing, reducing the possibility of bringing the virus into Luna Maya or back home. Additionally, between clients bedding is changed and rooms sanitized and ventilated.

Logistics involving water can complicate these protective measures. Water quality has deteriorated after two major earthquakes in 2017 had epicenters close to both Chiapas and Mexico City. Washing machines are common, but hot water washing is not. Propane gas is expensive; thus few homes have hot water connections for laundering. In warmer climates, the families in most homes forgo water heaters altogether, or in areas of less infrastructure, use wood burning options. At Luna Maya we have adapted by using healthy doses of detergent, a disinfectant in rinse cycles, additional time for line-drying (somewhat more complicated during the rainy season) and spacing of at least five days between uses of clothing or bedding. Midwives are welcome to leave worn items inside Luna Maya’s washing machines for laundering at the center or bring items home in a plastic bag, preferably sealed for 72 h prior to washing, to minimize dispersal of potentially contaminated particles. Low compliance with frequent clothing changes was becoming blatantly clear, especially at births where there is increased risk of transmission; group review of the RCM (2020) infographic of recommended PPE for obstetric and midwifery care contributed to better awareness about this measure. An additional measure to minimize fomite transmission is that midwives, staff and clients are removing shoes upon entering facilities—slippers have been placed at entrances—and it is recommended that midwives leave births with a fresh pair of shoes. Disinfectant spray is used on all items brought to births or home visits, both before and after, and when possible, washable cloth coverings are used on all surfaces of vehicles used to transport people and items.

### Screening and Scheduling Adjustments

Consideration for the local context has been especially necessary for screening and scheduling adjustments. Culturally, pregnancy and birth are considered family events. Our initial screening included asking women to arrive unaccompanied to all visits, yet, since Mexican culture is extremely family-oriented, women continued arriving with partners and other family members. Luna Maya is now asking each woman to limit her companions to only those she considers essential—a single person when possible; safe physical distancing during visits is our goal. This is especially important during births when midwives spend an extended period of time in the family home, and during postpartum visits, as we know that contact has typically been made with extended family. Arriving unaccompanied has been better accepted by women needing only well-woman care. The traditional Mexican greeting with a soft kiss on the cheek and a handshake has been eliminated nationally and at Luna Maya.

At staff meetings, we continually evaluate screening measures to determine their appropriateness; we have added questions about travel, atypical symptoms and contact with suspected cases. Barriers to midwifery care are multifaceted (Lopez Arellano et al., 2020) and the WHO's (2016) optimal number of 8 prenatal visits with providers exceeds what most Luna Maya families currently receive. Reducing and spacing visit days has been more feasible than reducing in-person perinatal contacts, and also provides time to clean, sanitize and ventilate facilities and rotate clothing. This scheduling change to fewer prenatal visits also minimizes days of possible exposure of midwives and staff to clients or while commuting.

### Distancing and “Telehealth”

All Luna Maya in-person classes and sessions—yoga, music for kids, childbirth preparation, and information sessions—were suspended immediately and providers offered support to continue online using a Zoom format. Middle- and upper-income families of Mexico generally have access to the internet at home, and either have laptops or smart phones for connectivity. Low income families generally do not have internet access at home but have mobile phones and frequently use WhatsApp for free communications. Therefore, video calls are available for all women seeking care at Luna Maya. We have also offered well-woman care not requiring physical assessment or evaluation online via Zoom or Whatsapp, while bodywork (massage, craniosacral treatments and any other body work), with the exception of acupuncture, has been postponed until it is safer to increase in-person visits. Virtual access has also facilitated access from other regions of Mexico and Latin America but has challenged continuity of care for those lacking a stable connection, electronic devices, or who simply prefer in-person care.

## Pandemic Stress: Changes in Birth as a Physiologic Process

We base this section on the themes that emerged from the qualitative interviews we conducted with eight Luna Maya midwives and apprentices. These interviews revealed how our midwives are responding to the increased stress experienced by childbearers while support options remain severely limited. Several midwives expressed concern that the current lack of in-person therapeutic options to alleviate stress-related body and emotional tension added to women feeling alone and may contribute to longer, more difficult labors:

*I feel further away. Some women get annoyed because of the safety measures and I try to find how to connect with them in other ways. The safety measures are there to create a barrier and keep people at a distance. They are designed to filter… to keep people out and they can be interpreted as a judgement. Midwifery is warmth, it’s being with and close to women. *(Luna Maya midwife, San Cristobal, Chiapas)**

Both the Luna Maya clinical staff and the larger Mexican midwifery community have noted a rise in abnormal birth patterns, leading to an increase in complications and emergencies. Through social media networks such as Facebook and Instagram, midwives practicing in Mexico both independently and in other birth centers report a greater number of hemorrhages and retained placentas, second stages that last 5 h or more, more babies that need resuscitation or CPR, and stalled labors that require transfer to the hospital—the one place our clients seek most to avoid during this pandemic:

*We see the [pandemic] effects before and after birth. The fact that they can´t get together with other moms makes things more difficult. Online support is fine, but the lactation consultants are not providing in-person care. In this context we see longer births, longer second stages, women fully dilated with no descent. During birth mammals need calm to release their baby.* (Luna Maya midwife, Mexico City)

*There are more post-term births because the babies are there, they have to be born but there is so much worry, there are blockages out of fear that things won't go well and that we have to go to a hospital. That adds more adrenaline.* (Student midwife, Luna Maya Mexico City)

Midwives expressed concern that some women may be choosing home birth exclusively out of fear of COVID-19 infection in hospitals and may not be fully prepared for OOH birth. At the beginning of the pandemic, women were switching to homebirth care late in their pregnancies in a context of generalized fear and uncertainty. Our midwife interlocutors noted that all this additional stress profoundly affects women's wellbeing and undermines the foundations of midwifery care:

*Some come into care at 37 weeks, so we have to hurry with visits and we don't have time for the childbirth education class, bodywork, and all of that has made it hard to establish a relationship of trust, [so] they make decisions based on fear. We had a bunch of transfers [to a hospital] in May, about 5 or so in a row. In two of them the women were 10 cm dilated. and the baby just wouldn't be born. There is so much additional uncertainty so much stress”* (Student midwife, Luna Maya Mexico City)

### Transfers to Hospital

During the prenatal process, women receiving care at Luna Maya decide whether they will be transferred to a public or private institution should the need arise. A vaginal or cesarean birth at a private hospital costs upwards of US$1000; thus the decision is often financial. Transfers to public hospitals often include no communication between the midwifery team and receiving medical team. Women are systematically bullied and scolded for having sought a midwife and a home birth; no support persons are allowed. Epidural for pain relief is not an option in public hospitals, where all women are placed on an oxytocin IV with no labor support from staff and are told to lie on their back with no option for movement. Most transfers to public hospitals end in cesareans. Although most such transfers are simply due to failure to progress, they are usually treated as dire emergencies by the medical staff, who often tell women their babies “were about to die” (see Davis-Floyd, 2003; Davis-Floyd, 2018).

In private hospitals, women can be accompanied by their partners and often by their midwife, who meets with the receiving obstetrician to go over case details. The outcomes of private transfers in Mexico City usually include vaginal birth with an epidural and oxytocin augmentation, whereas in Chiapas most transfers end in cesarean.

The transfers mentioned in the quote above were all due to failure to progress. One woman was transferred to a public hospital and had a cesarean birth. The remaining four were transferred to private hospitals and all but one had a cesarean. Due to COVID restrictions, only women´s partners were allowed into the birth setting after the transfer.

### Further Pandemic Stressors: The Curtailing of Women’s Rights, and Economic Impacts

Our interviews revealed the understanding among our Luna Maya clinical staff that the changes brought on by COVID-19 in maternity care practice are directly related to a world that does not support women and mothers in the first place and even less so during a pandemic. As has been the case during other global crises, women's rights and opportunities are often curtailed in times of instability:

*We’re seeing more violence against women in the home and everywhere. Families are not prioritizing a respected birth experience but are rather opting for a birth free of charge within the public health system, typically abundant with violence. Childbirth preparation class is also not a priority but covering basic living expenses and rent are. The economic constraints are very real—at the same time it seems absurd that more value is not being placed on out-of-hospital birth right now because it is a much safer option mid-pandemic.* (Student midwife, Luna Maya Mexico City)

The collective feeling of stress has also taken its toll on midwives: some of our colleagues or their families have contracted COVID-19, others have anxiety and/or have trouble sleeping. More work—because of women fleeing the hospital—in a stressful situation contributes to faster provider burnout, more clinical errors, more anxiety and less sleep. Our midwives who are also mothers have additional burdens. Children are home from school and workload has increased:

*There is tension sometimes. As a mom, I have more work because I spend more time with my daughter, it's a double workload for those of us who are mothers*. (Student midwife, Luna Maya Chiapas)

Another important theme that emerged is the dire economic impact on families who, prior to the pandemic, were already low-income and highly vulnerable. In particular, the Chiapas center clientele is 40% Indigenous and depends mostly on informal economy systems such as selling vegetables at the market, odd jobs such as cleaning and construction, and small family agriculture. Already this cohort was unable to pay a minimum fee for services, and Luna Maya Chiapas must rely on income from the Mexico City center and external funding to subsidize services in Chiapas. Unlike some US states (see Davis-Floyd et al., 2020), Mexico has not included community midwifery as an essential service during this pandemic nor does the government subsidize midwifery care, and clients must pay out-of-pocket. Thus midwifery care remains unreachable for many women because even though Luna Maya charges on a sliding scale, many families are unable to pay even minimal fees and thus have to birth in the public hospitals they fear, where care, though often violent and abusive, is free.

## Women's Experiences of Birth During COVID-19

This section is based on our interviews with eight women who were Luna Maya clients. We asked these women about their decision to give birth at home, how COVID-19 had impacted their daily lives and their pregnancy and birth experiences, their support networks and mental health, and their perceptions of the local and national health care system and its response to COVID-19, and received responses addressing all of the above. They also discussed how COVID-19 affected their sense of family and community and what this meant during pregnancy and postpartum, and pondered on the unmet needs of pregnant women in diverse, inequitable and changing contexts and in particular in terms of the capacity of the Mexican healthcare system to meet their needs.

### The Decision to Birth With Luna Maya

In the interviews, some women described that the decision to give birth with Luna Maya, at home or at the midwifery center, is often accompanied by previous knowledge of the Midwifery Model of Care and of the reality and outcomes of the Mexican healthcare system, and by the desire to avoid an unnecessary cesarean. As previously noted, Mexico holds one of the highest cesarean birth rates in Latin America, with almost 50% in public hospitals and over 80% in private institutions. Over 30% of women report having experienced obstetric violence (Castro and Frías, 2019). Our interlocutors expressed absolute clarity about their ability to give birth and the benefits of OOH birth, and about the level of safety and the professionalism of our midwives as their care providers. Women who were already suspicious of the national healthcare system confirmed that during COVID-19, their decision to birth outside of the hospital was appropriate and safe:

*Birth is instinctive. When we are in favorable conditions, when we feel held, when we feel secure, we can do it*. (Georgina, Mexico City)

A second pattern identified was women whose initial plan was to birth in the hospital because they felt safe there, yet sought out Luna Maya, mainly due to recommendations from other women. Learning about our model of care gave them reassurance, security and confidence, and they chose home birth so neither they nor their babies would be exposed to COVID-19 in hospitals:

*When all this COVID stuff started, we were going to the Social Security system [IMSS] for care. I went to make an appointment and I saw many elderly people sitting there coughing a lot and got really scared, because I thought I was putting my two-year-old daughter, myself and my baby at risk. That was when I decided to give birth at home as a better choice.It was very valuable and comforting to find Luna Maya, that they'd listen to our particular situation and to be able to be in constant contact. They are always there to help us and they do this job from the bottom of their hearts. *(Laura, Mexico City)

*That is one of the things that scares me, is going to see a doctor, because you still have to go to the hospital, and they are in contact with patients and other doctors. Of course they are national heroes, but they are also objects of danger. . .I'm sure that there are hundreds of women who are comfortable having their babies in the hospital, but I decided I wasn´t. Pregnant women are a high-risk group, but my mental health was most important*. (Carmen, San Cristobal)

When asked about Luna Maya´s Midwifery Model of Care, women highlighted the closeness, the welcoming feeling, the fact that they were emotionally sustained and treated well at all times. They also highlighted the importance of their partners being included, both during prenatal care and the birth and postpartum processes. In Mexico, birth partners are never permitted in public hospitals; during the pandemic, private hospitals varied on the level of accompaniment that was permitted. Women stated that they liked the attention the Luna Maya team gave to their personal circumstances, financial realities, lifestyle preferences, and adjustments due to the pandemic, and our curiosity about how they were holding up in their daily lives. One woman said:

*On the day I gave birth, I forgot everything. I threw COVID out the window, I held on to the midwives that were there with me, I don't remember right now if they were wearing facemasks. During labor I experienced it as if there were no COVID, as if they didn't have any protection. They were there, there was physical embrace, there were hugs, I am very grateful for that—that they conserve and make birth possible, the most respected birth possible*. (Georgina, Mexico City)

### The Effects of the Pandemic

All women interviewed expressed financial hardship due to the pandemic. The most critical situations were cases where women’s income depended on activities that were put on hold during lockdown and that, combined with pregnancy and birth, considerably augmented their stress, nervousness, sadness, irritability and above all, fear—a magnified fear that changed their lives radically:

*I am worried about when this will end and turn into a different lifestyle—the distance between people, the isolation, the question of how to bring up my daughter in this distance. It will end or more intense things will come*. (Laura, Mexico City)

The weight of financial difficulties not only affected these women’s stress levels, but also led families to make important lifestyle decisions that impacted daily life and family structure:

*The hardest has been the financial side. It has changed us, has altered everything, including something so simple as going to the market. I don't have a job… my husband's salary was cut by half. . . .It is hard to make sure that my daughter doesn't touch anything that comes from the outside. We changed as a couple, I was used to being alone and now we spend the whole day together. This family dynamic, and my daughter spending more time with her dad has its benefits, but it's only a little bit of light in the middle of so much complication. Sometimes I just feel really stressed out*. (Laura, Mexico City)

Many women are living this process with a generalized unprecedented fear—of getting infected, of infecting their babies and other children, their parents—and fear because they are a vulnerable population. They are constantly questioning themselves as to whether or not the measures applied in their daily lives are enough or if they are exaggerating about avoiding contact with friends and family:

*I lived through the transition [of care in Luna Maya before and during COVID]. I felt the difference. My family is far away, the only people with whom I felt understood and held were the midwives. I had a very intense pregnancy with many changes. I was relieved that I could relax and have my emotional needs taken care of. When contractions started and I encountered my midwives with masks, I felt once again that a wall had come up. I thought about the ambivalence, I had to think about being rational and taking precautions but. . . I wished that they could take it off*. (Georgina, Mexico City)

Women talked about the fact of living the pregnancy and childbirth process in ways they had never imagined, feeling generally excluded, isolated and without access to a support network. These feelings contrasted with the joys of having a new baby, yet sadness about the impossibility of sharing that joy coupled with additional sadness around C-19 deaths of people close to them:

*I have family members who died of COVID. We spent our moment of dreamy bliss to the opposing duality of death. That shook us deeply and filled us with fear*. (Georgina, Mexico City)

The theme of individual and collective fear came up in several interviews. Women talked about how the collective situation of mass disease impacted their daily decisions. They articulated the stress caused by the uncertainty and the constant state of alarm in both identifying symptoms in others and the collective fear of each other as potential disease vectors:

*I had never experienced fear like that. The first time we went out to vaccinate the baby, I came home and I changed all of my clothes because we had gone to the hospital. Just poking my head out on the street scared me so much, constantly listening to police cars, ambulances. I would get incredibly nervous. Fear was always present…This process has been about moving away from fear*. (Georgina, Mexico City)

The women spoke of the dichotomy of having more time with their families and themselves, while at the same time feeling inadequate as mothers to protect their children and explain the situation to them. Like so many of us, they tumbled around on the emotional roller coaster of isolation, yet with the added responsibility of going through the massive life transition of welcoming a new baby:

*We have a very strange psychology; we keep expecting to get it. My daughter got sick, she had a fever and sore throat, but no other symptoms. We have taken really good care of ourselves. It's really hard to explain to my daughter what is happening and that she can't play with other children. It has affected me. . .there are days where I feel really sad. And there are days where we are dancing in the living room and other days where you feel like… when will this day end, or you wake up with a feeling of “these four walls again.” One day I wanted to buy something from the supermarket, and they wouldn't let me in. Motherhood is complicated but managing everything we are going through in addition to our pregnancy is complicated and frustrating. There is a roller coaster of feelings. We can do things together, take a [virtual] yoga class or cook. Other days feel completely useless. We have lost certain freedoms. I question everything. This has affected our emotional health as a family*. (Carmen, San Cristobal)

### Positive Aspects of the Pandemic: Spending Time Together

Just as was expressed in the previous quote, our interlocutors were able to identify the positive aspects of being stuck at home and spending more time with their family. In particular, pregnancy is a time when women often yearn to be more alone and at home, and some identified this as an ideal opportunity for this psychological and physiologic response.

*COVID has given us the opportunity to be more connected to nature, to spend time together as a couple and process the pregnancy far from the noise and speed of the city. I would say that what really worried us was the decreased income, but even then, being outside of the city afforded us a more basic and simple lifestyle far from all the stimulus of the city*. (Georgina, Mexico City)

*I feel very calm. It has helped me very much to be at home. I used to work long hours at a restaurant. My routine changed very much. I can take better care of my diet and sleep. We are really enjoying this pregnancy, we have breakfast and dinner together, we share the same space… I've started to prepare the space [for when the baby comes]*. (Damaris, San Cristobal)

### Negative Aspects of the Pandemic: Isolation From Family and Friends

Mexico, as a Latinx country, relies heavily on family and social networks for meaning and ritual. Mexican women are accustomed to their mother, mother-in-law and close family members interfering, recommending, suggesting things, and caring for them during pregnancy and particularly in postpartum. It is customary for a woman´s mother or mother-in-law to move in with her during the six-week postpartum period to cook, tend to the home and other children and even prepare herbal baths and other rituals. In urban spaces, women have come to rely for support on the friendships made in childbirth education classes or in breastfeeding or childcare groups. Mexican society is highly social and highly family based, valuing the collective good over individual choice. Isolation from family, particularly elders, impacted women's experiences as it deeply challenged the meaning of the rite of passage of welcoming a new family member and the role of certain rituals for caretaking the mother:

*COVID stopped us from going to visit my family, and it made me feel very disconnected from those that I love, in a time when everything is so new, during pregnancy. I had felt the calling to be a mother in a certain type of world and COVID broke that world for me, leaving me wondering what will be of tomorrow*. (Samantha, Mexico City)

*It has affected our access to our support network… My in-laws are high risk. That means we cannot see them because traveling would put us and them at risk*. (Anabel, Mexico City)

Women described how their isolation from others affected their emotional health:

*Even though I am a person who likes privacy and space, I wanted to share this process, take it outwards and make it very social. I really wanted to experience this with family and friends… I feel like I have to hold things in more, that I have to do so much more, that I'm not being sustained emotionally… it makes me very angry*. (Laura, Mexico City)

*At the beginning I was going to yoga and childbirth education. I believe that [sharing and] being present with other moms gave us empathy for each other, that support of just going through the same things. With COVID we haven´t been able to have those support groups. As Mexicans we need those groups. I wasn't able to keep in contact with the other moms. I can imagine they feel the same way I do, they feel isolated and we need that in-person support network. Far from feeling taken care of, I feel rejected and excluded*. (Carmen, San Cristóbal)

*I started to feel the need to go out, to spend time with my family. They come and visit, but they stay on the patio and we talk through the gate. That is making me depressed because we haven't hugged, and my belly is growing and I am by myself.* (Damaris, San Cristóbal)

### Unmet Healthcare Needs of Pregnant Women

We asked women how their experiences during the pandemic should shape maternal health policy. They agreed on the need to acknowledge and integrate the Midwifery Model of Care and midwives as care providers within the Mexican healthcare system:

*[We need] the system to be more open to accepting the Midwifery Model in the healthcare system and for it to be supported with the necessary certifications and that the midwives be acknowledged. That could give us as clients the certainty to make decisions and approach the right people*. (Georgina, Mexico City)

They mentioned that there should be separate settings in or out of hospitals for prenatal visits and births to avoid exposing babies and adults to infectious diseases, describing the fear of putting their families at risk by visiting hospitals—a fear that compounds their perceptions of a healthcare system that already ignores the needs of women during pregnancy and birth:

*Every woman needs different care, not all women need the same thing, especially during the pandemic. Without the pandemic I think how they treat women is horrible, and during the pandemic they really should have separate spaces for women outside of the hospital, because in general it is very risky to go there*. (Alondra, San Cristóbal)

Women were clear in expressing that the pandemic came to exacerbate an already deficient healthcare system that fails to put women at the center of care.

*Mexico really doesn't have strategies that are adapted to our reality. They just take what they copy from elsewhere, they don't see the conditions of the majority of the population or of the groups they want to take care of. They are developed by someone who´s neither pregnant nor elderly nor at risk*. (Georgina, Mexico City)

*I think that more than ever we need psychological support and humanized care for pregnant women and during the postpartum. I think we have also seen that it wouldn't be a bad idea to have separate structures for providing prenatal and birth care. That way they could avoid exposing moms and babies to other risks. And I hope they didn't use this situation to keep up their cesarean section propaganda. They really need to let women have the right to choose how they birth*. (Samantha, Mexico City)

Women highlighted that improved support for their pregnancies did not involve more contact or visits with physicians; rather, it centered around the provision of information, connecting with other women, and ensuring emotional support. They stressed that some women have the privilege of accessing emotional support through the internet, but that many pregnant women in Mexico lack this resource:

*Especially information, sharing information that it's ok if we feel sad, tired even though I am pregnant, and this should be a happy time. Just letting us know that we might feel this way during the lockdown. Access to the internet has given me a support network to know that other women feel the same way I do. Other things like yoga, meditations… all on the internet, only that has saved us. There are women who don't have that possibility. They should share information, workshops with psychologists, doulas, that we aren't alone.* (Damaris, San Cristóbal)

## Discussion and Conclusions: Pandemic-Exacerbated Tensions

As experts in physiologic birth and sexual and reproductive health, midwives understand that these processes work best when they are spontaneous and surrounded by a calm and supportive environment. With the pandemic raging and lockdown encouraged, collective psycho-emotional health has suffered and pregnant women have been forced to add another stress factor to an already complex process. Even under normal circumstances, pregnancy, birth, and the postpartum period are transformational moments and uncertainty, doubts and fear make themselves present along the way. COVID-19 has increased stress levels, worries and anxiety; these increases in stress hormones have shown their impacts on the perinatal experience.

In this article, we have attempted to highlight that in a national healthcare system that for decades has failed to provide women the support needed for a healthy pregnancy—genuine emotional and psychological support and women-centered care—the pandemic has exacerbated these tensions. Midwives and researchers have long noted that birth is more than its medical definition as the extraction of a fetus from a uterus, and that healthcare systems are designed to place attention only on surviving that act. Such systems consistently alienate women and midwives, and establish a basis of mutual fear and mistrust.

For midwives, navigating the pandemic has meant an increased burden on how they do their work and how they navigate work-life balance. Midwives with children now have to figure out childcare during births to which they used to take their children, while providing emotional support in a socially distanced way. Forced to operate outside of the healthcare system, Luna Maya must rely on a fee-for-service pay structure, meaning that with the current economic crisis, our organization is faced with significant financial deficits. Yet we carry on, doing our best to provide our clients with the humanistic, woman-centered care they need, even in the face of a pandemic and a total lack of governmental support.

The call to action brought about by the COVID-19 pandemic has been that recommendations made by public health and clinical experts must be taken seriously and implemented immediately. All over the world, as shown in the other articles in this Special Issue, we are seeing strained systems exposing their vulnerabilities. Maternity health care in Mexico serves as yet another example of exposed vulnerabilities, and places added urgency on the need to build systems that support women's health, wellbeing, community-building and evidenced based care. If the shock and catastrophe of the COVID-19 pandemic does not bring action on decades of cries for improvement and woman-centered care, we may well wonder if anything ever will. We urge all governments and maternity-care-related organizations to seize this revelatory pandemic moment to improve maternity care practices, both nationally and globally.

## Data Availability

The raw data supporting the conclusions of this article will be made available by the authors, without undue reservation.
